# Pregnancy- and lactation-associated osteoporosis: A case series of 6 patients

**DOI:** 10.1097/MD.0000000000037430

**Published:** 2024-03-22

**Authors:** Ying Qian, Jingming Wang, Aifang Wu, Weimin Huang

**Affiliations:** a Department of Endocrinology, 960 Hospital of PLA, Jinan, Shandong, People’s Republic of China; b Department of Orthopedics, 960 Hospital of PLA, Jinan, Shandong, People’s Republic of China; c Department of Obstetrics, 960 Hospital of PLA, Jinan, Shandong, People’s Republic of China.

**Keywords:** case report, lactation, osteoporosis, pregnancy, vertebral fracture

## Abstract

**Rationale:**

There is still information about pregnancy- and lactation-associated osteoporosis, which is a type of osteoporosis that occurs in women with normal bone in the late pregnancy or lactation period.

**Patient concerns:**

Six cases of pregnancy- and lactation-associated osteoporosis diagnosed in our Endocrinology and Orthopedics Departments from January 2018 to June 2020 were retrospectively studied. The baseline characteristics, clinical features, laboratory findings, radiological manifestations, and follow-up outcomes were analyzed and compared with previous reports.

**Diagnoses:**

All six patients underwent magnetic resonance imaging scans and vertebral compressive fractures were detected in four patients.

**Outcomes:**

All six patients received conservative treatment and no surgical intervention. After a mean follow-up of 27.3 months (range 24–31 months), the symptoms of the six patients were significantly relieved, although four patients still had low back pain to varying degrees.

## 1. Introduction

Pregnancy- and lactation-associated osteoporosis (PLO) is a type of osteoporosis that occurs in women with normal bone in the late pregnancy or lactation period. It can manifest clinically as pain, bone loss, and even hip or back fracture.^[1,2]^ Due to the relatively rare occurrence of PLO, many clinicians lack knowledge of PLO, which prevents many patients from receiving timely diagnosis and appropriate treatment and results in various negative outcomes, such as postpartum chronic low back pain, vertebral compression fracture and kyphosis deformity.^[3]^ Also, as a result of the lack of awareness of the diagnosis and prognosis of PLO, some clinicians even perform unnecessary surgical interventions, like vertebroplasty, internal fixation or hip replacement, which causes irreversible iatrogenic damage and is a waste of medical resources.^[4,5]^

Therefore, it is extremely important to promote the knowledge and understanding of PLO among clinicians in the departments of orthopedics, endocrinology and obstetrics. However, there is currently limited information on PLO. A systematic review has been conducted to examine the risk factors, clinical manifestation, and treatment methods for this disease,^[6]^ but it should be noted that there is still a lack of related research on PLO. This study describes the clinical features, treatment and prognosis of 6 PLO patients admitted to our hospital and aims to enhance the understanding of this rare disease.

## 2. Methods

### 2.1. Study design

Patients diagnosed with PLO in our hospital from January 2018 to June 2020 were retrospectively studied. Enrolled patients were excluded if they had been diagnosed with other secondary osteoporotic diseases, like hyperparathyroidism, Cushing syndrome, and hyperthyroidism. Ethical approval was obtained from the ethics committee at our hospital.

### 2.2. Data collection

Baseline characteristics, individual medical history, medication history, family history, pregnancy and childbirth history, laboratory tests, and neonatal characteristics were recorded retrospectively. The detailed information was demonstrated in Table 1.

**Table 1:** Baseline characteristics.

Case	Age (yr)	Height (cm)	Weight before pregnancy(kg)	Fracture history	Smoking history	Menstruation history	Medication history (glucocorticoids, heparin, anticonvulsants)	Family history of osteoporosis
1	35	168	49	No	No	13, normal	No	No
2	36	163	59	No	No	12, irregular	No	Mother
3	30	157	49	No	No	12, normal	No	No
4	33	171	60	No	No	11, irregular	No	Mother
5	34	160	73	No	No	11, normal	No	No
6	33	157	47	No	No	13, normal	No	No

### 2.3. Bone mineral density measurement and diagnostic criteria

Dual-energy X-ray absorptiometry (DXA) examination of lumbar spine (L2–L4) was used for bone mineral density (BMD) measurement on a Norland XR-600 Bone Densitometer (Norland Medical System, Inc., Fort Atkinson, WI). All the examinations were performed on the same machine and software in the Radiology Department of our hospital. The diagnostic criteria were as follows: patients had osteoporotic fracture or low back pain in late pregnancy or early postpartum with a *Z* value of −2 or lower, as evaluated by a DXA examination of lumbar spine.^[7]^

## 3. Results

Six patients with an average age of 33.5 years (30–36 years), all of whom were of Han ethnicity, were included in this study. Their average height was 163 cm (range 157–171), average weight was 56.2 kg (range 47–73), and average body mass index was 21.3 kg/m^2^ (range 17.4–28.5). Among the common risk factors previously reported in the literature, 2 patients had a family history of osteoporosis, none of the patients had a history of smoking, a history of prepregnancy fracture, or a history of oral anticoagulant and glucocorticoid use (Table 1).

All patients were naturally conceived and had no history of assisted reproduction. Five patients had onset during the first pregnancy and 1 patient during the second pregnancy. The fetal sex was 2 males and 4 females. Five were breastfed postoperatively (Table 2).

**Table 2:** Pregnancy and delivery characteristics.

Case	Assisted reproduction	Pregnancy and delivery history	Production time at onset	Weight gain during pregnancy (kg)	Delivery mode	Fetal sex	Fetal weight (kg)	Feeding mode
1	No	G1P1	1	15	Natural labor	Female	3.0	Breast feeding
2	No	G3P2	2	21	Natural labor	Male	3.5	Breast feeding
3	No	G1P1	1	10	Natural labor	Female	2.7	Breast feeding
4	No	G1P1	1	16	Cesarean section	Female	3.5	Breast feeding
5	No	G1P1	1	10	Cesarean section	Male	2.9	Artificial feeding
6	No	G2P2	1	20	Natural labor	Female	2.9	Breast feeding

All the cases presented as back pain, and the average visual analog scale score was 7.0 (range 4–10 points). The onset of pain in 6 patients was within the third trimester and 3 months postpartum. The detailed information is shown in Table 3.

**Table 3:** Clinical features.

Case	Time of back pain onset	VAS at visit	Time of fracture	Fracture site	BMD*	25-VD (ng/mL)	PINP (ng/mL)	β-CTX (μg/L)	BGLAP (μg/L)	PTH (pg/mL)	Treatment	Follow-up time (mo)	VAS at the final follow-up
1	1 month before delivery	5	2 months postpartum	T11, L2	−2.87	13.42	125	1.14	39.4	43	Calcium and VD	31	2
2	3 months before delivery	6	No fracture	No fracture	−2.67	26.49	81.8	0.325	22.1	13	Ceasing breast feeding, calcitonin, calcium and VD	22	3
3	3 months before delivery	10	3 months postpartum	T12, L2, L5	−4.37	22.64	74.4	2.19	18.1	31	Ceasing breast feeding, calcitonin, calcium and VD, teriparatide	26	2
4	1 month post delivery	8	4 months postpartum	T11, T12, L1	−1.34	21.24	102	0.843	25.8	28	Ceasing breast feeding	30	3
5	3 months before delivery	4	No fracture	No fracture	−2.11	NA	NA	NA	NA	NA	Calcium and VD	24	0
6	2 months after delivery	9	4 months postpartum	T6-T8, T11-L4	−3.27	5.35	131	0.909	37.2	24	Ceasing breast feeding, calcium and VD, menatetrenone	31	0

25-VD = 25-hydroxyvitamin D, BGLAP = bone gamma-carboxyglutamate protein, BMD = bone mineral density, CTX = type I collagen C-terminal peptide, PINP = procollagen I N-terminal extension peptide, PTH = parathyroid hormone, VAS = visual analog scale.

* BMD was evaluated by the *Z* value of dual-energy X-ray absorptiometry examination on lumbar spine.

### 3.1. Laboratory tests

Bone remodeling markers including procollagen I N-terminal extension peptide, type I collagen C-terminal cross-linked telopeptide, bone gamma-carboxyglutamate protein, parathyroid hormone, and 25-hydroxyvitamin D_3_ were measured in 5 patients. The mean values of procollagen I N-terminal extension peptide, C-terminal cross-linked telopeptide, bone gamma-carboxyglutamate protein, parathyroid hormone, and 25-hydroxyvitamin D_3_ were 103 ng/mL (range 74–131), 1.08 µg/L (range 0.34–2.19), 28.5 µg/L (range 18.1–39.4), 28.0 pg/mL (13–43), and 17.9 ng/mL (range 5.4–26.5), respectively.

### 3.2. BMD and radiographic features

**Figure 1.**
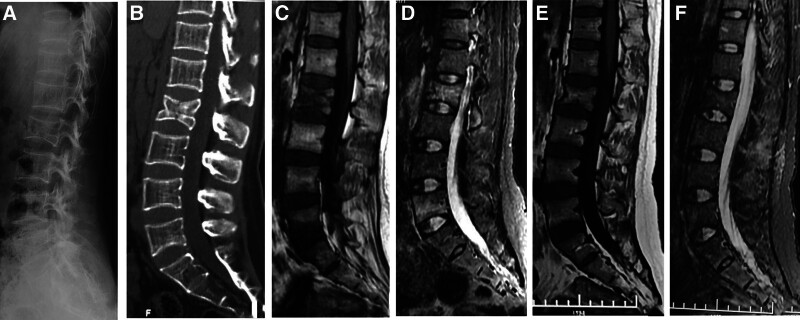
(A) The lateral X-ray film of the patient shows the compression change in L2 vertebral body on admission. (B) CT images reveals compression change and abnormal density in the L2 vertebral body on admission. (C) T1-weighted MRI shows abnormal low signal changes in the L2 vertebral body, lower edge of L5 vertebral body, and upper edge of S1 vertebral body on admission. (D) T2-weighted fat-suppressed MRI shows abnormal high signal changes in the L2 vertebral body, lower edge of L5 vertebral body, and upper edge of S1 vertebral body on admission. (E) After 3 months of conservative treatment, T1-weighted MRI shows that the abnormal signal in the affected vertebral body disappeared; (F) after 3 months of conservative treatment, T2-weighted fat-suppressed MRI shows that the abnormal signal in the affected vertebral body has disappeared. CT = Computer Tomography, MRI = magnetic resonance imaging.

The average *Z*-score obtained from the DXA measurement of lumbar spine BMD was −2.90 (range −4.37 to 1.34). All 6 patients underwent magnetic resonance imaging (MRI) examination, and four of them had vertebral compression fractures. The main feature of MRI images of the patients was a strip-shaped abnormal signal area under the upper endplate of the vertebral body, with low T1 phase signal and high T2 fat-suppressed phase signal. Fractures were mainly distributed in the thoracolumbar region (Fig. 1), and the 4 patients with vertebral compression fractures had multiple vertebral fractures with at least 2 levels and at most 9 levels.

### 3.3. Treatment and prognosis

All patients received conservative treatment and no surgical intervention. Oral calcium and vitamin D were administered to all 6 patients as baseline treatment and 4 patients were required to cease breastfeeding by doctor’s advice. Two patients received short-term salmon calcitonin, 1 patient received teriparatide, and 1 patient received bisphosphonates. During the mean follow-up period of 27.3 months (range 24–31), the symptoms were obviously relieved in all patients, but 4 patients still had low back pain to varying degrees. One patient carried her second pregnancy to term without suffering from PLO again.

## 4. Discussion

Since PLO was first reported by Nordin in 1955, little epidemiological data on PLO has been reported.^[8]^ Smith^[9]^ reported that the incidence of PLO was approximately 4–8 cases per million persons. A previous systematic review of 338 cases worldwide revealed that the average age of PLO patients was 35.7 years old and the average body mass index was 22.2 kg/m^2^. Ninety-two percent of patients developed clinical symptoms between 3 months prior and postdelivery.^[6]^

In recent years, case reports and case series of PLO have gradually increased due to increasing awareness of this disease, but there is still little documented research PLO in China. The authors of these reports speculate that there might be a potentially higher incidence of PLO in China due to the adherence of Chinese people to the traditional custom of postpartum confinement.^[10]^ Postpartum confinement means that the mother must strictly rest in a sealed room at home for 1 month immediately after giving birth. During the postpartum confinement period, the mother is not allowed to go out or open the window for ventilation. In Chinese traditional practice, postpartum confinement could help women recover better. However, the lack of exposure to ultraviolet sunlight may have a negative effect on the synthesis of vitamin D and bones, and increase the incidence of PLO.^[11]^

Many studies have shown that PLO is a multifactorial disease with unclear pathogenesis. It has been suggested that genetic and endocrine factors play an important role in the development of PLO. Mutations in low-density lipoprotein receptor-related protein 5 have been found to be significantly associated with PLO.^[12–14]^ Low-estrogen status after long-term anorexia, stress, excessive exercise and even premature ovarian failure, have been documented to be correlated with PLO.^[15]^ Some studies have shown that increased calcium requirement for fetal skeletal development may also contribute to the development of PLO. At the end of pregnancy, fetal bone formation consumes about 30 grams of calcium, and more than 80% of calcium deposition occurs in the last trimester, which causes a sharp loss of maternal calcium and might lead to PLO during this period.^[16,17]^ In addition, low calcium intake, vitamin D deficiency, and use of glucocorticoids, heparin, and some anticonvulsants during pregnancy have also been suggested as risk factors for PLO.^[18–20]^

Fractures may occur in patients with severe PLO, and the most commonly involved site is the lumbar or thoracic spine region.^[21,22]^ MRI is the most important examination to establish a definitive diagnosis. A typical MRI feature is strip-shaped abnormal signal area adjacent to the endplate in hypointense T1 and hyperintense T2 fat-suppressed phase.^[23]^ It should be noted that different from postmenopausal osteoporotic fractures, PLO vertebral fractures often involve multiple vertebral fractures. In the present study, all PLO patients suffered vertebral fractures involving multiple vertebral fractures. This is consistent with the results of a previously published systematic review, reporting an average of 4.4 vertebral fractures among the 155 enrolled patients.^[6]^ In view of this imaging feature, it is recommended that all PLO patients with vertebral fractures routinely undergo both lumbar and thoracic MRI examinations at the same time to rule out concurrent thoracolumbar fractures.

At present, there is still no consensus on the treatment of PLO, and the treatment protocol is mainly referred to that of postmenopausal osteoporosis. It is generally accepted that breastfeeding should be discontinued immediately after the establishment of a PLO diagnosis. Individualized drug treatment should be provided according to pain severity, bone density, bone remodeling markers, and prepregnancy plan.^[24]^ Currently, the commonly used drugs include calcium and vitamin D, calcitonin, bisphosphonate, teriparatide, and denosumab, etc. Calcium and vitamin D can still be used as basic treatment. For patients with severe pain, calcitonin drugs can be considered due to its better analgesic effect in the short-term.^[25]^ Bisphosphonate has been widely used in the treatment of postmenopausal osteoporosis. However, since the drug will deposit in the bone tissue for a long time due to its long half-life, it remains unclear whether bisphosphonate will have an impact on the fetus during the second pregnancy. Therefore, for patients who may choose to have a second pregnancy, bisphosphonate should be carefully used.^[26,27]^ Teriparatide has a shorter half-life compared with bisphosphate and has been reported to have good clinical effect on PLO.^[28,29]^ Denosumab is also widely used and has achieved good results in the treatment of PLO. Although its half-life is longer than that of teriparatide, it is more convenient to use and has better compliance.^[30]^

Although the symptoms in all 6 patients were relieved at the last follow-up date in this study, there were still 4 patients with residual low back pain of varying degrees, suggesting that the symptoms of PLO may take a long time to disappear. Kyveritakis evaluated the clinical prognosis of PLO patients during a 6-year follow-up period, and found that 58% of the patients needed more than 3 years for the complete relieve of clinical symptoms, and about 1/4 of the patients suffered refracture. These findings indicate that PLO may not be a simple self-limiting disease, and requires appropriate therapy.^[21]^

## 5. Conclusion

PLO tends to occur in older and thinner women with clinical manifestations of varying severity. MRI often reveals multiple vertebral compressive fractures. The prognosis with conservative treatment is good, but complete relief may take a long time.

## Acknowledgments

The authors would like to thank the investigators and patients who contributed to this study.

## Author contributions

**Conceptualization:** Weimin Huang.

**Data curation:** Ying Qian, Jingming Wang, Weimin Huang.

**Formal analysis:** Aifang Wu.

**Investigation:** Ying Qian, Jingming Wang, Aifang Wu.

**Methodology:** Jingming Wang.

**Resources:** Aifang Wu, Weimin Huang.

**Supervision:** Jingming Wang, Weimin Huang.

**Writing – original draft:** Ying Qian.

**Writing – review & editing:** Aifang Wu, Weimin Huang.
